# A new survival model for hyperthermic intraperitoneal chemotherapy (HIPEC) in tumor-bearing rats in the treatment of peritoneal carcinomatosis

**DOI:** 10.1186/1471-2407-5-56

**Published:** 2005-05-30

**Authors:** Joerg OW Pelz, Joerg Doerfer, Werner Hohenberger, Thomas Meyer

**Affiliations:** 1 Department of Surgery, University of Erlangen-Nuremberg, Germany

## Abstract

**Background:**

Cytoreduction followed by hyperthermic intraperitoneal chemotherapy (HIPEC) improves survival in patients with peritoneal carcinomatosis of colorectal origin. Animal models are important in the evaluation of new treatment modalities. The purpose of this study was to devise an experimental setting which can be routinely used for the investigation of HIPEC in peritoneal carcinomatosis.

**Methods:**

A new peritoneal perfusion system in tumor bearing rats were tested. For this purpose CC531 colon carcinoma cells were implanted intraperitoneally in Wag/Rija rats. After 10 days of tumor growth the animals were randomized into three groups of six animals each: group 1: control (n = 6), group 2: HIPEC with mitomycin C in a concentration of 15 mg/m^2 ^(n = 6), group III: mitomycin C i.p. as monotherapy in a concentration of 10 mg/m^2 ^(n = 6). After 10 days, total tumor weight and the extent of tumor spread, as classified by the modified Peritoneal Cancer Index (PCI), were assessed by autopsy of the animals.

**Results:**

No postoperative deaths were observed. Conjunctivitis, lethargy and loss of appetite were the main side effects in the HIPEC group. No severe locoregional or systemic toxity was observed. All control animals developed massive tumor growth. Tumor load was significantly reduced in the treatment group and was lowest in group II.

**Conclusion:**

The combination of hyperthermia with MMC resulted in an increased tumoricidal effect in the rat model. The presented model provides an opportunity to study the mechanism and effect of hyperthermic intraperitoneal chemotherapy and new drugs for this treatment modality.

## Background

Peritoneal carcinomatosis (PC) represents an advanced form of cancer and is associated with a poor prognosis and quality of life. The peritoneal failure rate among patients who present with recurrence after colon cancer resection is approximately 25–35% [1]. The median survival time after manifestation of peritoneal carcinomatosis is about 6 months. If peritoneal carcinomatosis is present, normaly there is no curative treatment available for any of the tumors in the abdominal cavity.

Hyperthermic intraperitoneal chemotherapy (HIPEC) is a new treatment modality. Complete cytoreductive surgery plus HIPEC is effective in the prevention and treatment of peritoneal metastases and it should lead to long-term survival for serosa-invasive carcinoma patients [2]. The efficacy of intraperitoneal (i.p.) chemotherapy is synergistically by hyperthermia. Although the technique of hyperthermic intraperitoneal chemoperfusion in humans has been employed in cancer therapy, controlled studies using this approach are rare. Intraperitoneal chemotherapy results in a high local drug concentration with less systemic exposure compared to conventional i.v. drug administration [3,4]. Peritoneal lavage with mitomycin C (MMC) results in a drug exposure of the peritoneal surface that is 20 times higher than elsewhere in the body [5,6]. The additional toxic effect of MMC at temperatures higher than 39°C has been demonstrated both in animals and in *in vitro *models [7,8].

One disadvantage of HIPEC is the limited penetration depth of cytostatic drugs into the tissue [4]. Thus, HIPEC must be combined with cytoreductive surgery. However, complex surgical techniques in an experimental setting should be comparable to the clinical situation. We therefore developed a miniaturised animal model and herewith report the details of the design and its efficiency.

## Methods

### Animals

18 inbred male pathogen-free WAG/Rija rats weighing 200 to 240 g, obtained from Charles River, Sulzfeld, Germany, were used in this study. The animals were kept in individual cages during the experiment and 12 hours of light per day. They were fed a standard laboratory diet and tap water *ad libidum*. Maintenance and care of all experimental animals were carried out according to the guidelines of the local responsible Animal Protection Commission and carried out in compliance with national guidelines (National Institute of Health for Use of Laboratory Animals).

### Tumor

The tumor cell line used was a moderately differentiated adenocarcinoma of the rat colon (tumor cell line CC531; 1,2-dimithylhydrazine-induced [9]) obtained from the German Cancer Research Centre, Heidelberg, Germany.

Intraperitoneal tumor application was performed with a tumor suspension produced in vitro. The tumor cell line was cultivated at 37°C and 5% CO_2 _in monolayer cultures in an incubator in 20 ml complete medium (RPMI 1640 [Gibco, Life Technologies, Eggenstein, Germany], 10% heat-inactivated fetal bovine serum [Seromed, Biochrom, Berlin, Germany] and 1% Pen/Strep [Seromed]). After three days, cells were washed twice with phosphate buffered saline (PBS) and were detached with 3 ml trypsin (0.25 %). Trypsin was deactivated by adding the complete medium. After centrifugation, washing and re-suspension with PBS, vitality was evaluated in a Bürker hematocytometer after the addition of trypan blue. After vital counting, the suspension had a density of 2,5 × 10^6 ^vital cells/200 μl suspension (after centrifugation and re-suspension in PBS) before being injected into the animals. To control for possible mutations of cell lines, only cultures that had undergone less than 10 passages were used in the experiments.

The tumor cells were implanted via laparotomy performed under general anesthesia. The rats were anaesthetized by using isoflurane. The abdomen was shaved and cleaned with 70% alcohol. The laparotomy was performed using a lower middle incision of 6 cm. The tumor cell suspension was injected under the capsule of the peritoneal surface in the right upper side of the abdomen. Hence, tumor size and localisation were comparable to the human situation, especially after extensive cytoreductive surgery (Fig 3).

The animals were randomised into three groups of six animals each: group 1: control (n = 6), and group 2: HIPEC with MMC in a concentration of 15 mg/m^2 ^(n = 6), group 3: MMC i.p. in a concentration of 10 mg/m^2 ^(n = 6).

**Figure 1:**
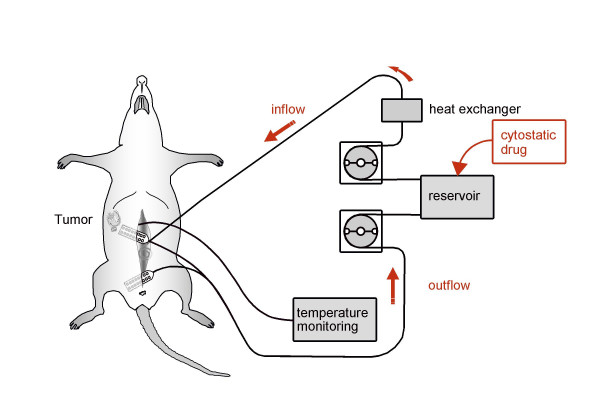
Diagram of the experimental HIPEC circuit.

The equipment consisted of a miniature heat exchanger and a roller pump. The peritoneal perfusate was warmed in the heat exchanger (which consists of two outer silicone tubes with diameter of 15 mm for the in- and out-flowing water (Fig. 1)). The warmed perfusate was driven by a roller pump with two synchronously running pump-heads on a single axis for the inflow and the outflow lines (Masterflex^®^).

The intraperitoneal temperature was maintained between 40.5 and 41.5°C. Perfusion was performed over 90 minutes after the perfusion fluid had reached the required temperature. In group II, mitomycin C was added to the perfusate in three divided doses at 30 min intervals in a drug concentration of 15 mg/m^2^. The body surface of the animals was calculated according the formula (A(m^2^) = m_k_^0,425 ^× 1_K_^0,725^/139.315). The first dose contained 50% and the following administrations 25% of the total dose.

For temperature measurement during perfusion, a nickel-chrome-nickel thermocouple of 0.6 mm in diameter (Standard Integrated Thermocouple Thermocoax, Phillips, Hamburg, Germany) was placed near the macroscopic tumor margin. Temperature was also monitored in the rectal cavity. The thermocouple was calibrated before use in a high-precision water bath. Baseline temperature was recorded for 5 minutes before treatment. Temperature was continuously measured during application. In group I (control) and group III (MMC only) temperature measurement was not performed.

After the perfusion the perfusate was removed and the abdomen was irrigated with saline for 10 minutes. Thereafter the abdomen was closed in two layers.

### Evaluation

All animals were kept in individual cages. The rats were weighed and inspected for side effects (lethargy, loss of appetite, fatigue syndrome, wound infection) daily. The animals were kept under standard conditions and were euthanised by an overdose of anesthetic and cervical dislocation 10 days after treatment.

The animals were autopsied and peritoneal carcinomatosis was evaluated qualitatively and quantitatively. The tumor nodules were counted macroscopically.

### Tumor response

Tumor response was graded according to the classification system as described by Steller et al. [10]. The scoring ranged from 0 to 5 and was performed by 2 independent observers. A score of 0 meant that there was no tumor growth; a score of 1 indicated an estimated tumor diameter less than 0.5 cm; a score of 2, a tumor diameter between 0.5 and 1 cm; a score of 3, a tumor diameter between 1 and 2 cm; a score of 4, a tumor diameter between 2 and 3 cm; and a score of 5, a tumor diameter of more than 3 cm.

### Statistical Analysis

Data were analyzed using SPSS/PC+ statistical software. The mean scores were calculated. The significance of differences was assessed by the Kruskal-Wallis-test. A p value < 0.05 was considered to be significant.

## Results

All animals survived the operative procedure and could be evaluated 10 days following surgery. No unexpected death occured after surgery.

### Perfusion characteristics

Intraoperatively the required tissue temperature was reached within 9–11 minutes in the HIPEC group. Stable temperatures were then maintained for a further 90 minutes with a mean of 40.4°C ± 0.5. The volume needed to fill the perfusion circuit was about 250 ml. To reach tissue temperature the perfusion fluid was maintained at 45,3°C ± 2.3. The calculated body surface of the animals was 0.03–0.04 m^2^.

**Figure 2:**
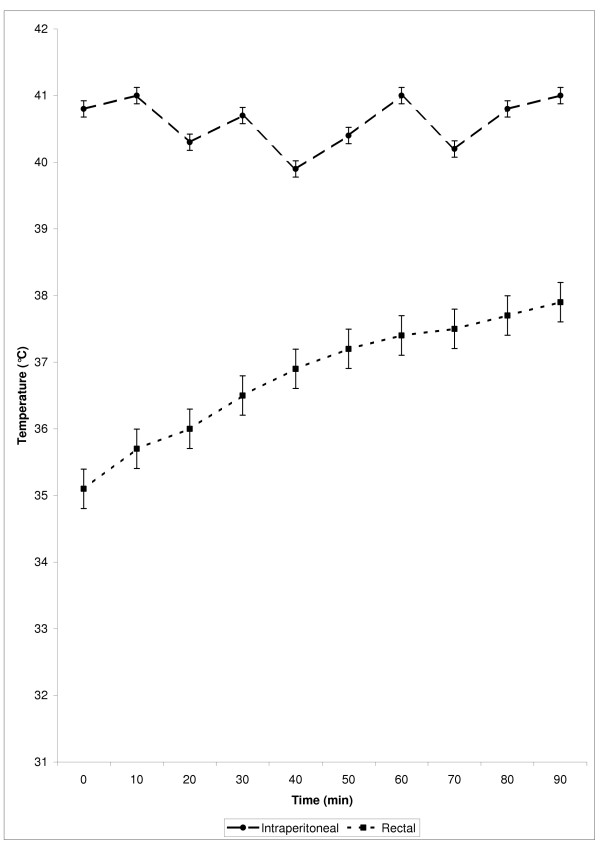
The temperature course during HIPEC in the abdomen and in the rectal cavity.

**Figure 3:**
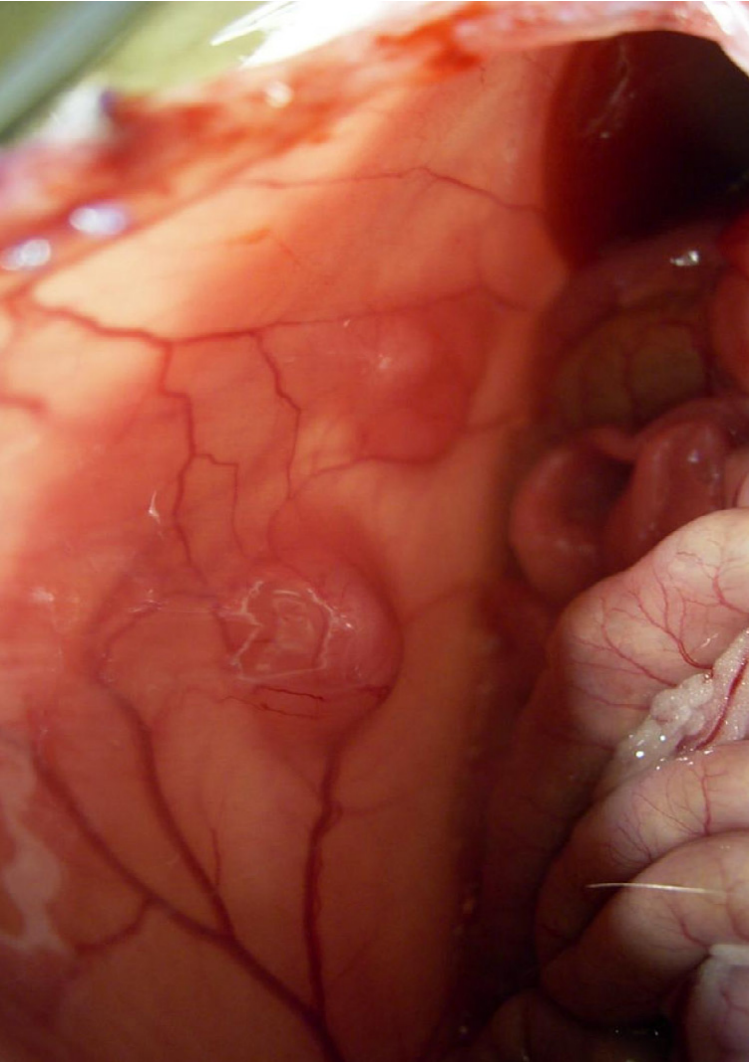
Limited tumor implantation. Injection of cell suspension under the capsule of the peritoneal surface preventing diffuse peritoneal carcinomatosis (a). Isolated tumour nodule without diffuse carcinoses 10 days after implantation (b). Representative gross sections of peritoneal carcinomatosis in group I (control), II (HIPEC) and group III (MMC only) 10 days postoperatively.

The course of intraperitoneal and rectal temperatures are presented in Fig. 2.

### Duration of the procedure

The operation time, including the time needed to install the perfusion circuit, varied from 112 to 135 min (mean 121) in group II.

### Body weight

Postoperatively body weight decreased in all groups during the first 5 days with a maximum decrease of 5 per cent. In the following 3 days, body weight recovered up to 108 percent of the pre-operative level. No significant differences were found between the experimental groups.

### Clinical appearance

Conjunctivitis, lethargy and loss of appetite were the main side effects observed in each of the treatment groups. These were particulary pronounced in the HIPEC group. After 10 days 4/6 animals in group II had minor bleeding in the peritoneum. Moderate toxic reactions of the peritoneum were found in two animals in group III (MMC i.p.). Control animals had no side effects in the gastrointestinal tract.

### Macroscopic tumor growth

In group I, all animals developed extensive intraperitoneal tumor growth. The median tumor weight was 8,1 ± 3,4 g. In group II and III median tumor weight was 1,8 ± 0,9 g and 5,7 ± 2,4 g. The cancer indices were significantly lower in group III (2,6) than in group I (3,5). The lowest tumor load was observed in group II (1,4). Tumor nodules in group II were 4 ± 2. Tumor nodules in group II were significant more after 10 days (35 ± 12) The tumor scores are given in table 1.

In group I, liver metastases were observed in two of the rats.

## Discussion

Hyperthermic intraperitoneal chemotherapy (HIPEC) with mitomycin C has been applied following cytoreductive surgery for various peritoneal surface malignancies. Spratt et al. first performed HIPEC in a patient with pseudomyxoma peritonei [11]. A significant survival benefit has been shown for HIPEC when compared to systemic chemotherapy alone [4]. The complete cytoreductive surgery is the most important prognostic factor. Incomplete cytoreduction results in limited survival [12,13].

The goal of this study was to investigate the feasibility of HIPEC surviving tumor-bearing rats model and to investigate the efficacy of HIPEC applied to a single tumor growing intraperitoneally.

Hyperthermia and drugs used during HIPEC procedures have a limited penetration depth [14,4]. Therefore, HIPEC can only be effective in patients with minimal residual disease after extensive cytoreductive surgery. In the majority of related animal studies, tumor cells were injected directly into the abdominal cavity. This procedure results in diffuse growth of peritoneal carcinomatosis. To simulate cytoreductive surgery in our animal model, the CC531 colon carcinoma was implanted in an intraperitonael fat pad as described by Veenhuizen et al. [15]. Using this technique, we prevented diffuse tumor spread in the abdominal cavity. This technique may better simulate the clinical situation after de-bulking. The development of the tumors was 100 %. In contrast, Los described that only 80% of rats developed carcinomatosis after i.p. inoculation [3,4].

HIPEC could be conducted without difficulty in all cases. None of the animals died during perfusion. During and after 10 days following intervention, no serious complications were observed. In contrast to the control group (no treatment), there were 3 cases of mild side effects in group II. However, all of these side effects were completely reversible after a few days. During the procedure the abdominal cavity was kept open, allowing the abdominal perfusate to be stirred, resulting in a more homogeneous distribution of heated drug solution throughout the entire abdominal cavity.

The fact that an average temperature (of the perfusate) of 45,6°C was required in order to ensure the necessary intraabdominal temperature of 40,5°C, demonstrates the high degree of cooling that occurred across the large surface area. This question the feasibility of a closed abdomen procedure, which has been both postulated and clinically practised. An additional benefit of such a procedure would be a reduction of the potential MMC exposure risk for the operating staff. However, a major concern associated with a closed abdominal procedure is the inhomogenous distribution of the cytostatic drug and the intraabdominal temperature, because the abdominal peritoneal perfusate cannot be manually stirred as in open perfusion. A homogenous distribution of temperature is reliably assured by the open approach only, together with measurement of the temperature directely at the tumor, i.e. the target site.

MMC was chosen as the chemotherapeutic agent because of its known direct cytotoxic effect in the treatment of colorectal cancer and the thermal enhancement of its activity [16]. Heated intraperitoneal MMC is used in the clinical HIPEC setting with a concentration of 30–35 mg/m^2 ^[17]. This concentration is 50 % higher than the maximum tolerated intravenous dose of MMC (20 mg/m^2^) because HIPEC results in decreased drug absorption from the perfusate (approx. 70%) [5]. Compared to the clinical situation, the MMC ratio in our experimental trial was 1,5:1 (HIPEC versus i.p. therapy). In animal models, MMC proved highly potent in the experimental setting and completely prevented intraperitoneal tumor growth in the maximal tolerated dosis of 20 mg/m^2 ^i.p. [18]. In comparison, the MMC concentrations chosen for the study were lower than the maximal tolerated dose, but also administered in a ratio of 1,5:1 (15 mg/m^2 ^HIPEC versus 10 mg/m^2 ^i.p.). Our results showed that MMC concentration of 10 mg/m^2 ^was not effective enough to completely inhibit tumor growth in group III (therapy of solid tumor) in contrast to the study of HRIBASCHEK et al. (preventing of peritoneal carcinomatosis)(18).

Primary results regarding tumor response in this HIPEC animal model confirm the clinical data collected thus far. In comparison to the control group, a significant delay of tumor growth was demonstrated in group III. This shows that MMC, as a monotherapy, has a clear influence on tumor response. The fact that tumor growth was merely slowed (but not arrested) demonstrates that the chosen MMC concentration was not too high.

## Conclusion

By the presented model, the procedure of HIPEC could be evaluated qualitatively and quantitatively. The direct effect of HIPEC could be recorded macroscopically. Long-term observations can be conducted without difficulty, assuming acceptable mortality and morbidity rates.

In conclusion, intraperitoneal macroscopic tumor growth was reduced after hyperthermic intraperitoneal chemoperfusion in comparison with MMC application alone. However, further experimental *in vitro *and *in vivo *studies are required to better charaterize the exact mechanism of HIPEC and to set up objective parameters for optimization of the procedure.

## Competing interests

The author(s) declare that they have no competing interests.

## Authors' contributions

JP carried out the treatment and drafted the manuscript.

JD carried out the treatment.

WH participiated in the design of the study

TM participiated in the design and coordination of the study.

All authors read and approved the final manuscript.

## Pre-publication history

The pre-publication history for this paper can be accessed here:



## Figures and Tables

**Figure 4:**
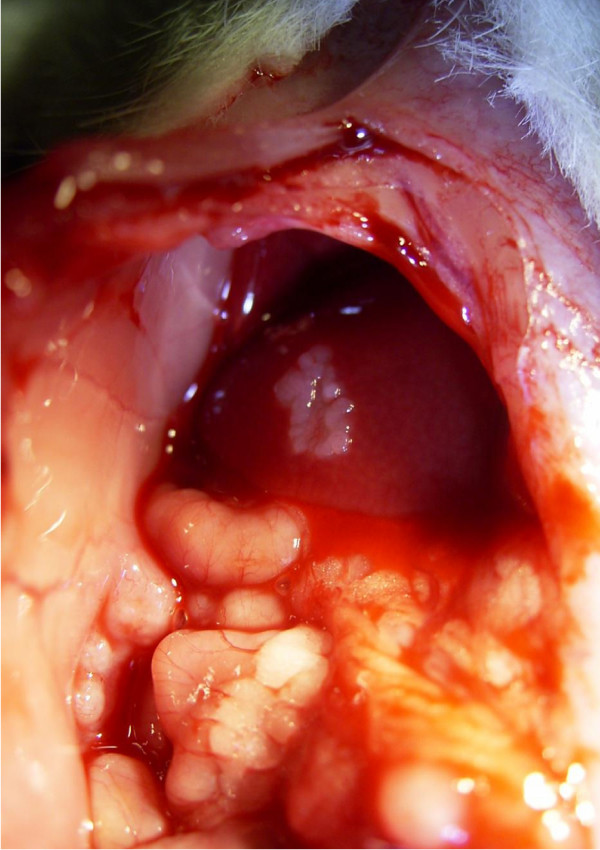
Diffuse intraperitoneal tumor spread in the control group (group I).

**Figure 5:**
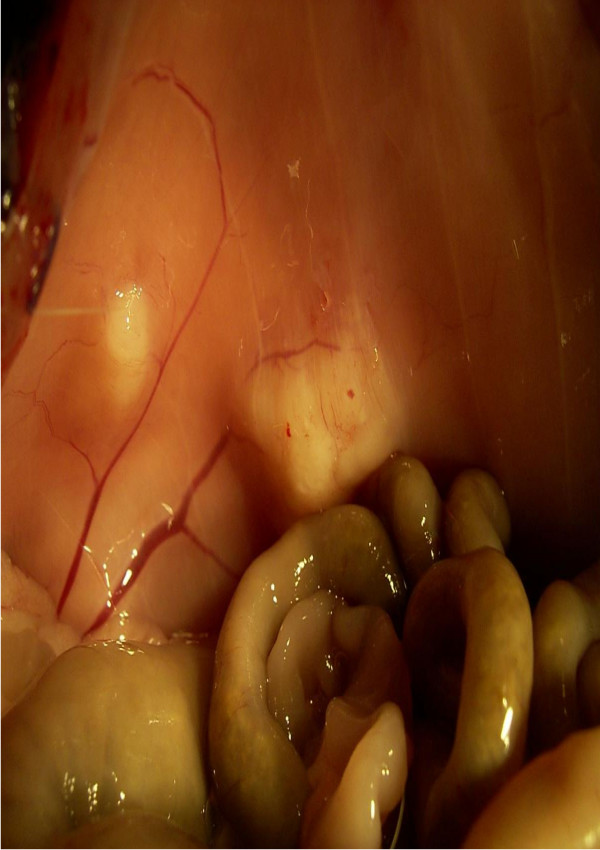
Small tumor load in HIPEC group (group II).

**Figure 6:**
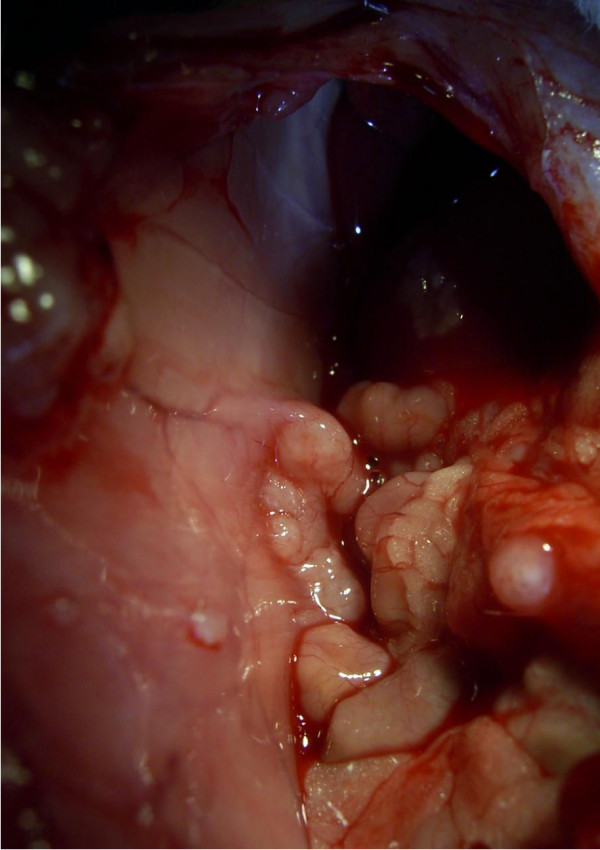
Tumor growth after MMC application (group III).

**Table 1:** Objective tumor response in groups 1 (control; n = 6), 2 (HIPEC; n = 6), and 3 (MMC only; n = 6) 10 days after intervention. * P < 0.05 versus groups 1 and 3; § P < 0.05 versus group 1 (Kruskal-Wallis test)

	Group I	Group II	Group III
Tumor weight (g)	8,1 ± 3,4	1,8 ± 0,9 *	5,7 ± 2,4 ^§^
Tumor nodules	35 ± 12	4 ± 2 *	16 ± 10 ^§^
Cancer index	3,5	1,4 *	2,6
Ascites	2/6	0/6	0/6
Clinical CR	0/6	4/6	1/6
